# The TGF-β Pathway: A Pharmacological Target in Hepatocellular Carcinoma?

**DOI:** 10.3390/cancers13133248

**Published:** 2021-06-29

**Authors:** Ester Gonzalez-Sanchez, Javier Vaquero, Maite G. Férnandez-Barrena, Juan José Lasarte, Matías A. Avila, Pablo Sarobe, María Reig, Mariona Calvo, Isabel Fabregat

**Affiliations:** 1 Centro de Investigación Biomédica en Red de Enfermedades Hepáticas y Digestivas (CIBEREHD), National Biomedical Research Institute on Liver and Gastrointestinal Diseases, Instituto de Salud Carlos III, 28029 Madrid, Spain; jvaquero@idibell.cat (J.V.); magarfer@unav.es (M.G.F.-B.); maavila@unav.es (M.A.A.); psarobe@unav.es (P.S.); mreig1@clinic.cat (M.R.); 2 Bellvitge Biomedical Research Institute (IDIBELL), L’Hospitalet de Llobregat, 08908 Barcelona, Spain; mcalvo@iconcologia.net; 3 Department of Physiological Sciences, Faculty of Medicine and Health Sciences, University of Barcelona, L’Hospitalet de Llobregat, 08907 Barcelona, Spain; 4 Hepatology Programme, CIMA-University of Navarra, 31008 Pamplona, Spain; 5 Instituto de Investigaciones Sanitarias de Navarra IdiSNA, 31008 Pamplona, Spain; jjlasarte@unav.es; 6 Immunology and Immunotherapy Programme, CIMA-University of Navarra, 31008 Pamplona, Spain; 7 Barcelona Clinic Liver Cancer (BCLC) Group, Liver Unit, Hospital Clinic Barcelona, August Pi i Sunyer Biomedical Research Institute (IDIBAPS), University of Barcelona, 08036 Barcelona, Spain; 8 Oncología Médica, Institut Català d’Oncologia, L’Hospitalet del Llobregat, 08908 Barcelona, Spain

**Keywords:** TGF-beta, TGF-beta inhibitors, HCC, HCC immunotherapy, HCC targeted therapy

## Abstract

**Simple Summary:**

Transforming Growth Factor-beta (TGF-β) signaling is crucial to maintain tissue homeostasis. Alterations in TGF-β signaling impact tissue functions and favor the development of diseases, including cancer. In hepatocellular carcinoma (HCC), the most frequent liver tumor, TGF-β plays a dual role, acting as a tumor-suppressor at early stages but contributing to tumor progression at late stages. TGF-β can also act on the stroma, favoring progression and driving immune evasion of cancer cells. Therefore, inhibiting the TGF-β pathway may constitute an effective option for HCC treatment. However, its inhibition in the wrong patients could have negative effects. To overcome this obstacle, it is mandatory to identify relevant biomarkers of the status of TGF-β signaling in HCC. In this review we summarize the functions of TGF-β in HCC and the available strategies for targeting TGF-β signaling. We also present the clinical results of the use of TGF-β inhibitors and their future in HCC.

**Abstract:**

Transforming Growth Factor-beta (TGF-β) superfamily members are essential for tissue homeostasis and consequently, dysregulation of their signaling pathways contributes to the development of human diseases. In the liver, TGF-β signaling participates in all the stages of disease progression from initial liver injury to hepatocellular carcinoma (HCC). During liver carcinogenesis, TGF-β plays a dual role on the malignant cell, behaving as a suppressor factor at early stages, but contributing to later tumor progression once cells escape from its cytostatic effects. Moreover, TGF-β can modulate the response of the cells forming the tumor microenvironment that may also contribute to HCC progression, and drive immune evasion of cancer cells. Thus, targeting the TGF-β pathway may constitute an effective therapeutic option for HCC treatment. However, it is crucial to identify biomarkers that allow to predict the response of the tumors and appropriately select the patients that could benefit from TGF-β inhibitory therapies. Here we review the functions of TGF-β on HCC malignant and tumor microenvironment cells, and the current strategies targeting TGF-β signaling for cancer therapy. We also summarize the clinical impact of TGF-β inhibitors in HCC patients and provide a perspective on its future use alone or in combinatorial strategies for HCC treatment.

## 1. Introduction

Hepatocellular carcinoma (HCC) is the most frequent form of primary liver cancer, the sixth most diagnosed cancer and the fourth leading cause of death by cancer. Despite its death burden, there are limited efficient therapeutic options against it. Surgery (liver transplantation or tumor resection) may be the major curative option but is only available for patients with early-stage cancer. Substantial advances have been made in the last years in understanding the central events that drive malignant transformation and progression, as well as in the systemic therapies approved for the treatment of unresectable HCC [1,2,3]. Recent emerging data of clinical studies showed manageable toxicity and safety for immunotherapeutic approaches [4], although limited therapeutic benefit. One urgent issue is how to convert liver cancer from cold to hot and responsive. One ongoing approach is to design combinatorial treatment of different immune checkpoint inhibitors with other reagents and modalities, as recently approved for HCC [5]. Pathways involved both in immunosuppression and cancer development and progression represent interesting therapeutic targets for liver cancer. In this regard, the Transforming Growth Factor-beta (TGF-β) pathway [6] represents an excellent candidate. However, its role in hepatocarcinogenesis is complex. This review will focus on the role of TGF-β in liver cancer, with the main aim of better understanding whether, or not, TGF-β inhibitors may represent a promising combinatorial therapy in HCC.

## 2. TGF-β Signaling

TGF-β superfamily consists of 33 multifunctional cytokines, including TGF-βs, activins, inhibins, bone morphogenetic proteins (BMPs) and growth and differentiation factors (GDFs). TGF-β, the prototypical member of the family, presents three isoforms in mammals (TGF-β1, TGF-β2 and TGF-β3), among which TGF-β1 is the most abundant and well-studied. TGF-β signaling plays key roles in the regulation of different cellular processes, including proliferation, differentiation, migration or cell death, which are essential for tissue homeostasis. Consequently, dysregulation of its pathways contributes to human disease.

**Figure 1:**
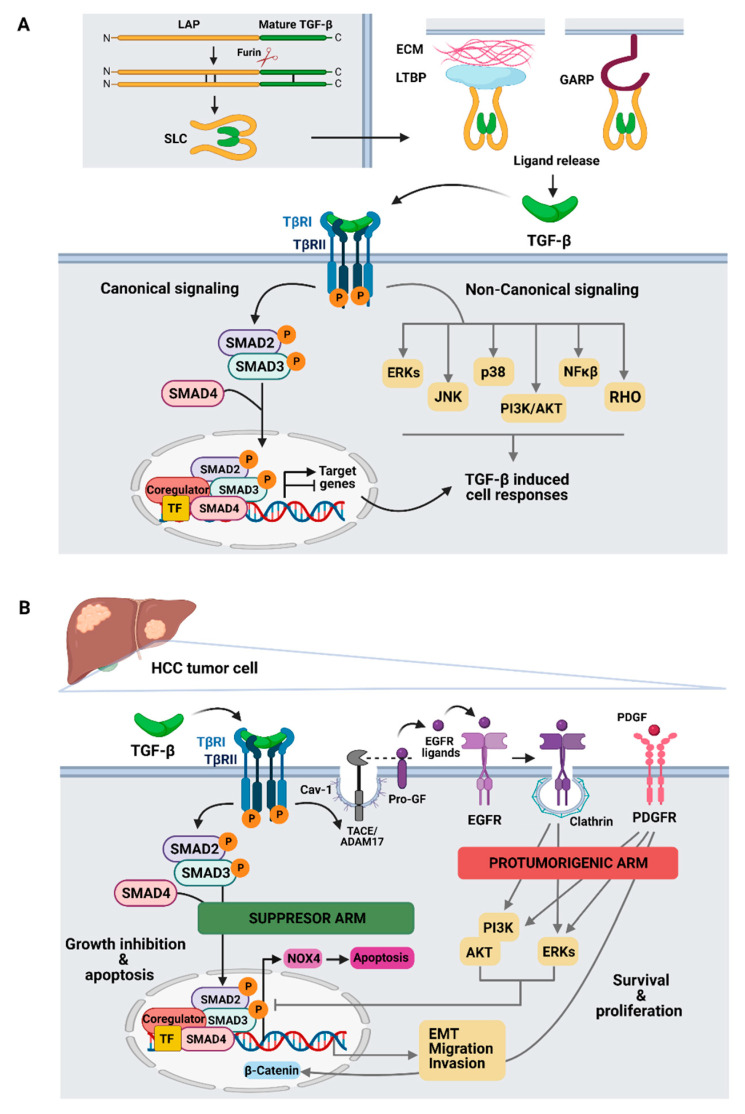
TGF-β-mediated signaling in liver cells. (**A**) Canonical (SMAD-dependent) and non-canonical (non-SMAD) signaling pathways. (**B**) TGF-β dual role controlling tumor suppressor and protumorigenic responses in hepatocellular carcinoma. Cav-1: Caveolin, ECM: extracellular matrix, GF: Growth Factor, LTBP: latent TGF-β binding proteins, SLC small latent complex, LAP: Latency associated peptide. Figure was created with BioRender.com.

Nearly all cells, including those of the liver [7], produce and secrete TGF-β (Figure 1A). First, TGF-β is synthesized in the rough endoplasmic reticulum as a pro-peptide precursor consisting of a large N-terminal pro-segment named Latency Associated Peptide (LAP) and a C-terminal mature polypeptide (mature TGF-β). Next, pro-peptide precursor’s dimers are processed in the Golgi network by the furin convertase to form a small latent complex (SLC) in which the LAP portions shield the mature TGF-β, preventing it from binding to its receptors. Finally, SLC are secreted and deposited in the extracellular matrix (ECM) by bonding with the latent TGF-β binding proteins (LTBPs) or anchored to the cell surface by glycoprotein-A repetition predominant protein (GARP) [8] (Figure 1A). Although different mechanisms may account for the activation of mature TGF-β, the integrins αv-mediated process, leading to the release of the LAP by contractile forces, appears to have a predominant role [8,9].

Once released, mature TGF-β binds to TGF-β type I and type II serine/threonine kinase membrane receptors (i.e., TβRI and TβRII) triggering the formation of an heterotetrameric complex in which the constitutively activated type II receptor phosphorylates and activates the type I receptor. After the extracellular signal is transduced across the membrane, activated TβRI initiates the canonical TGF-β signaling pathway through the phosphorylation of the Receptor-regulated (R)-SMADs 2 and 3 in their C-terminal serine residues. Thereafter, phosphorylated SMAD2 and 3 form a trimeric complex with a common mediator (Co)-SMAD4 and translocate into the nucleus where they need to interact with other transcription factors to activate or repress the transcription of target genes [10] (Figure 1A). Besides transcriptional regulation, interaction of SMADs with specific transcription factors and co-regulators enables them to regulate gene expression by alternative mechanisms including epigenetic remodeling, RNA splicing and miRNA processing [10]. 

In addition to the SMAD pathway, TGF-β can also initiate multiple non-SMAD or non-canonical signaling pathways (Figure 1A) [10,11]. For example, due to its weak tyrosine kinase activity, TβRI can induce the phosphorylation of Src homology domain 2-containing protein (Shc) and subsequently activate the ERK MAP kinase pathway. Besides, TβRI can also recruit TGFβ-activated kinase 1 (TAK1), through tumor necrosis factor-associated factor (TRAF) 4 or 6 to stimulate JNK, p38 MAPK and NF-κB pathways, or activate RHO small GTPases leading to actin cytoskeleton reorganization, whereas TβRII can directly phosphorylate the cell polarity regulator PAR6. AKT signaling can also be activated by TGF-β in a PI3K dependent matter. Moreover, these signaling pathways may also cross-talk with the TGF-β canonical signaling through post-translational control of SMAD activation and functions [10]. 

Due to its pleiotropic effects, TGF-β’s activity needs to be carefully controlled to maintain tissue homeostasis. Multiple mechanisms may account for this strict regulation including: the actions of (i) sequestering proteins that prevent TGF-β binding to its receptors; (ii) accessory coreceptors such as TβRIII (betaglycan), which regulate the presentation of ligands to the TβRII/TβRI receptor complexes; or (iii) inhibitory proteins as SMAD7, which antagonize the activation of SMADs 2 and 3; (iv) the control of TGF-β response by cell surface distribution of TβR receptors and (v) post-translational modifications of TβR and SMADs, such as phosphorylation, ubiquitination and sumoylation [9].

In the liver, TGF-β plays a major role in physiological and pathological conditions. Expression of TGF-β ligands is increased in chronic liver diseases and TGF-β signaling participates in all the stages of disease progression from initial liver injury to HCC [12,13,14,15]. In cancer settings, alterations in TGF-β signaling both in the malignant cells and the tumor microenvironment (i.e., cancer associated myofibroblasts (CAF), endothelial cells and immune cells) may contribute to the progression of HCC as thoroughly described below. 

## 3. Role of TGF-β in HCC Cells

Expression of TGF-β ligands is increased in liver chronic diseases and TGF-β signaling participates in all stages of disease progression [12]. In liver carcinogenesis, TGF-β plays a dual role, behaving as a suppressor factor at early stages, but contributing to later tumor progression once cells escape from its cytostatic effects. In non-transformed hepatocytes, TGF-β inhibits proliferation [16] and induces apoptosis [17,18]. Activation of TGF-β signal induces antiproliferative signals in epithelial cells through SMAD-dependent transcriptional regulation of genes that codify for proteins involved in cell cycle, such as Retinoblastoma, Cyclin- Dependent Kinase (CDK) inhibitors, or c-Myc, among others [19,20,21]. Apoptosis induced by TGF-β also requires SMAD-dependent transcription, although other non-canonical signals have been proposed to be involved. In hepatocytes and liver tumor cells, TGF-β-induced apoptosis requires up-regulation of the NADPH oxidase NOX4 (Figure 1B) that mediates reactive oxygen (ROS) production, which is required for regulating mitochondrial-dependent cell death [22,23,24]. 

Taking all this under consideration, it should be expected that the TGF-β pathway plays a tumor suppressor role in liver cancer. However, malignant cells surpass the suppressive effects of TGF-β either by inactivation of key components of the pathway or through the overactivation of parallel pathways that counteract its suppressive effects, such as production of autocrine factors, included EGFR ligands or PDGF and their receptors (Figure 1B) [25,26]. The autocrine loop of EGFR activated by TGF-β in HCC cells requires activation of the metalloprotease TACE/ADAM17 located in caveolin compartments in the cell membrane [27]. Moreover, clathrin (*CTLC*) has been recently identified as a key regulator of TGF-β-mediated EGFR transactivation in this context [28]. Thus, up-regulation of EGFR ligands and activation of EGFR signaling enhances the capacity of the cells to overcome the pro-apoptotic effects of TGF-β. In late stages, tumor cells that have acquired resistance to TGF-β suppressor functions respond to it acquiring capabilities that contribute to tumor progression. Indeed, HCC cells respond to TGF-β by inducing phenotypic changes related to a full or a partial Epithelial-Mesenchymal Transition (EMT), that contribute to increase the tumor cell migratory and invasive capacities and confer them properties of a migratory tumor initiating cell [29,30]. Taking together these and many other studies, it is very clear that TGF-β plays a dual role in the progression of HCC. 

An elegant study from Coulouarn and col. proposed different liver TGF-β signatures in HCC cell lines and patients defining a cohort of genes related to its tumor suppressor capacities, which they designed as the “early signature” and another cohort of genes related to its tumor promoting effects, the “late signature” [31]. The early signature pattern correlated with longer and the late signature response pattern with shorter survival in HCC patients. In addition, tumors expressing the late gene signature displayed invasive phenotype, increased tumor recurrence and accurately predicted liver metastasis. Increased TGF-β levels and mutations in the key molecules involved in the TGF-β pathway have been found in a relevant percentage of HCC patients. In this sense, somatic mutations in at least 1 gene whose product is a member of the TGF-β pathway have been found in 38% of HCC samples [32]. Some of them correlated with loss of TGF-β tumor suppressor activity, but other ones were related to overactivation of the TGF-β pathway, which contribute to amplify its pro-tumorigenic, pro-inflammatory and pro-fibrotic actions. Molecular gene signatures reflecting the TGF-β oncogenic arm have also been identified in tumors across the different HCC molecular classification [33]. 

Indeed, targeting the TGF-β pathway may be a promising therapeutic option in HCC, but it is necessary the identification of biomarkers that help to identify which is the response of the tumor cells in patients. In this sense, in vitro and in vivo studies and analyses in patients have identified *CXCR4*, *CD44*, *SMAD7* or *CLTC* as genes that are upregulated by TGF-β in the HCC tumor cell, correlating with its pro-tumorigenic arm. The high expression of these genes, together with high expression of TGF-β1, may help to identify patients with a “late TGF-β signature” that would benefit from TGF-β targeting drugs [28,30,34,35,36,37,38]. Table 1 compiles the information regarding the expression of the above-mentioned biomarkers in the human HCC cells lines for which more published information can be found, according to their TGF-β signature. Recent pharmacological studies with Galunisertib, a TβRI kinase inhibitor, are also allowing the identification of biomarkers that may help to calculate the benefit and/or to follow up the potential efficiency of TGF-β blockers in the progression of HCC [39].

## 4. TGFβ-Related Functions in HCC Tumor Microenvironment (TME)

**Figure 2:**
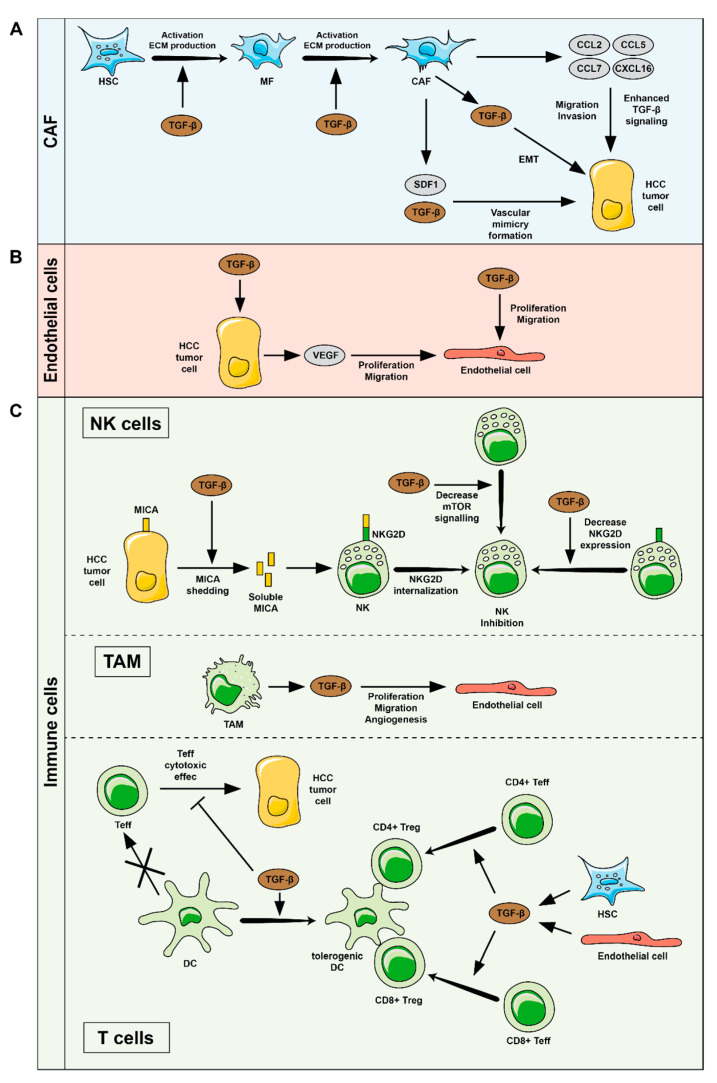
Schematic representation of the effects of TGF-β on the stromal cell types of hepatocellular carcinoma. (**A**) Effects on CAF: TGF-β stimulates the activation of HSC and the maintenance of the myofibroblastic phenotype, which, upon malignant transformation of hepatocytes into HCC cells, become CAF. CAF produce (i) TGF-β, that acts on HCC cells inducing EMT, and enhance vascular mimicry formation in HCC cells together with SDF1, (ii) other chemokines (CCL2, CCL5, CCL7 and CXCL16) that enhance TGF-β activity on HCC cells leading to metastasis. (**B**) Effects on endothelial cells: TGF-β may promote migration and proliferation of endothelial cells by (i) directly acting on endothelial cells or (ii) inducing VEGF secretion by HCC cells. (**C**) Effects on the immune system: TGF-β may increase the levels of soluble MICA and weaken the action of natural killer (NK) cells through its binding to NKG2D. TGF-β can also downregulate NKG2D expression or repress the mTOR pathway impairing tumor recognition. TGF-β can also act on tumor associated macrophages (TAM) that secrete growth factors and promote migration of endothelial cells and angiogenesis. TGF-β can favor the generation of tolerogenic dendritic cells (DC) and impair the activation of effector T lymphocytes and their cytotoxic activity. TGF-β promotes the conversion of conventional CD4 or CD8 T cells into immunosuppressive Treg cells that can hinder the antitumor activity of the adaptive immune response. CAF: cancer associated fibroblasts, ECM: extracellular matrix, EMT Epithelial-Mesenchymal Transition, HSC: hepatic stellate cells, MF: myofibroblasts, Teff: effector T cells, Treg: T regulatory cells.

The tumor microenvironment (TME) consists of a variety of resident and infiltrating host cells, secreted growth factors and cytokines, and ECM proteins that provide a scaffold for the infiltration and migration of the different cellular components inside the tumor, including tumor cells. Through reciprocal interactions with malignant cells, stromal cells (CAF, endothelial cells and immune cells) contribute to the accumulation of ECM, angiogenesis, inflammation, metastasis, and the suppression of the anti-tumorigenic adaptive immune cell response. During hepatocarcinogenesis, TGF-β is produced by most cell types and takes part in the dialogue between tumor cells and host stroma, placing it as a key player in the regulation of these important hallmarks of cancer progression as detailed hereafter (Figure 2).

### 4.1. HSC and CAF

HCC usually develops from a background of chronic liver disease that in most cases curses through premalignant states of fibrosis and subsequent cirrhosis, which provide the proper environment for hepatocyte malignant transformation [40]. One of the hallmarks of liver fibrosis is the activation of hepatic stellate cells (HSC) to myofibroblasts, which in turn triggers the transformation of the microenvironment by producing ECM deposits and releasing pro-fibrotic and pro-inflammatory factors that contribute to chronic liver disease progression [40]. In fact, most studies agree that, in the liver, the major sources of myofibroblasts in experimental models of fibrosis are HSC [41,42,43,44,45]. Eventually these activated myofibroblasts will evolve to CAF during hepatocarcinogenesis, although other sources, such as the differentiation of recruited bone marrow derived mesenchymal cells or the transformation of epithelial cells through EMT, that have been described in other cancers [46], cannot be entirely ruled out. 

The master role of TGF-β in the activation of HSC to myofibroblasts during liver fibrosis has been profoundly studied (detailed revisions on this subject can be found [47]). Briefly, TGF-β stimulates the activation of HSC and the maintenance of the myofibroblastic phenotype [42,48,49]. This effect is mediated through the activation of canonical (SMAD3) [12,50,51,52] and non-canonical (ERK, JNK, p38, and STAT3) [53,54] intracellular signaling pathways that induce the expression of pro-fibrogenic genes, including *COL1A1* (encoding collagen 1) and *CCN2* (encoding CTGF). In turn, CTGF stimulates the production of ECM components. In addition, NOX4, by the production of ROS, has been described as a central mediator of the TGF-β-induced activation of HSC [49,55].

As in the fibrotic tissue, in the TME the activation of CAF supports tumor progression by producing ECM and cytokines, stimulating immune evasion, and promoting angiogenesis [46]. However, despite the extensive information on the role of TGF-β in HSC activation in the fibrotic liver or CAF generation in other cancers [56], the studies related to TGF-β and CAF in HCC are scarce. The best-known effects of TGF-β on CAF consist of the induction of a pro-fibrotic phenotype, which is negatively regulated by LXRα through the interaction of this nuclear receptor with SMAD3 at the *ACTA2* promoter sites [57]. Furthermore, LXRα antagonistic effects were able to impair the pro-tumorigenic effects of CAF on malignant HCC cells in a 3D co-culture model [57]. But CAF not only respond to TGF-β, they also produce and secrete this cytokine affecting the crosstalk between tumor and stromal cells (Figure 2A). In this sense, CAF-secreted TGF-β in conjunction with SDF1 has been shown to enhance the expression of VE-cadherin, MMP2 and laminin5γ2, leading to vascular mimicry formation in HCC cells in a mechanism negatively regulated by miR-101 [58]. Moreover, CAF secrete multiple chemokines (CCL2, CCL5, CCL7 and CXCL16) that promote HCC cell migration and invasion through enhancing TGF-β activity in HCC cells, leading to HCC metastasis [59]. Indeed, a study modelling tumor-stroma interaction revealed that TGF-β secreted by HSC and myofibroblasts can mediate EMT in HCC cells [60].

Despite these advances, much is still to elucidate about the role of TGF-β in HCC CAF. For instance, CAF autocrine TGF-β has been associated in other cancers to the recruitment of more CAF and the subsequent deposition of high amounts of ECM. These deposits of ECM increase tumor stiffness reducing the density of blood vessels, thus forming a barrier that can impair the access of anti-cancer drugs and immune cells to the tumor [61,62,63,64]. In the same direction, recent advances in single cell sequencing techniques have allowed the characterization of distinct CAF subpopulations with different specific functions, such as CAF subtypes with high α-SMA expression, that demonstrate a strong TGF-β responsiveness [65,66,67,68]. Further characterization of HCC CAF subpopulations could shed light on the role of TGF-β in CAF and other cell types in the TME, providing opportunities for their specific targeting.

### 4.2. Endothelial Cells

Angiogenesis is a hallmark of cancer progression as it facilitates tumor growth and metastasis. Endothelial cells lining newly formed blood vessels nourish tumors and mediate the entry and exit of immune cells and other substances. TGF-β has been demonstrated as a pro-angiogenic factor in different tumors. In HCC, TGF-β secretion by mesenchymal stem cells was related to an increased angiogenesis in mouse models [69]. Another study linked the angiogenic effects of TGF-β secreted by HCC cells to the signaling mediated by its coreceptor CD105 [70]. Indeed, endothelial cells isolated from HCC showed higher expression of CD105 and enhanced capacity to migrate in response to TGF-β than normal endothelial cells [70] (Figure 2B). In HCC patients, the worst malignant features were correlated with the highest expression of TGF-β, CD105 and angiogenic markers [70]. Thus, inhibition of TGF-β signaling using LY2109761 was able to reduce vessel formation in tumors but showed no effects on physiological angiogenetic development [70]. On the other side of this crosstalk, exposure to TGF-β induces production of VEGF in HCC cells in a mechanism mediated by TGF-β/SMAD3/NF-κB signaling cascade [71] (Figure 2B). Consequently, VEGF actions on endothelial cells ultimately led to promotion of angiogenesis. The same study showed that treatment with arsenic trioxide impaired this effect by upregulating miR-491, which directly targets the expression of SMAD3.

### 4.3. Immune System

The liver is the largest peripheral immunomodulatory organ and is filled with a multitude of innate and adaptive immune cells, including Kupffer cells, natural killer (NK) cells, NK T (NKT) cells, and liver-transiting and/or -resident CD8+ T cells and CD4+ T cells [72]. TGF-β critically regulates immune cells in the liver to maintain a balance between immune tolerance and activation. This immune homeostasis is required to properly control inflammatory processes and prevent autoimmune alterations. But this equilibrium can be altered by TGF-β released within the TME, which may promote cancer progression through differential effects on multiple key cell types that orchestrate innate and adaptive immunity.

As it is well known, immune TME is heterogeneous. The HCC immune landscape shows that approximately 25% of cases have a high degree of immune infiltration (so-called ‘immune class’ of HCC), with high expression levels of PD-1/PD-L1 [73,74,75] and likely well suited for PD-1-blocking immunotherapy. Notably, intratumoral immune cell densities of CD3+ and CD8+ cells associate significantly with recurrence and relapse free survival [76,77]. The HCC immune class was further subdivided into an active immune response subtype (65% of immune class samples), characterized by overexpression of adaptive immune response genes, and an immune exhaustion subtype (35%), characterized by the presence of immunosuppressive signals and cells (TGF-β and M2 macrophages). Importantly, patients in the active immune response cluster showed improved survival and lower rates of tumor recurrence compared with patients in the exhausted immune response cluster. This study allows the development of classification tools for HCC patients to predict their response to immunotherapy or to design new therapeutic strategies [75]. A strong association between the TGF-β signature and the exhausted immune signature in HCC was identified, suggesting that the TGF-β pathway is an important immune regulator and biomarker for HCC [32,74,75]. In fact, several types of innate and adaptive immune cells respond to TGF-β released by cancer cells, stromal cells and immune cells themselves, resulting in an immunosuppressive TME (Figure 2C).

TGF-β in innate immune cells in HCC: Natural killer cells (NK) are part of the first line of immunological defence against cancer development. Defects in NK cell numbers and functions are recognized as important mechanisms for immune evasion of tumor cells in HCC [78]. NK cell function appears to be attenuated in HCC and various mechanisms seem to be involved in their malfunction. On the one hand, TGF-β directly inhibits the activation and functions of NK cells by repressing the mTOR pathway [79]. On the other hand, TGF-β, directly or through post-transcriptional mechanisms, controls the expression of the activation receptor NKG2D in NK cells [80]. Intra-tumoral NK cells have NKG2D downregulation in comparison to NK cells in non-tumor liver [81]. Furthermore, some tumor cells also downregulate NKG2D ligands, such as MICA, on the tumor cell membrane. The increase of soluble MICA by the action of MICA-shedding proteases has been reported in several patients with HCC, impairing the action of NK cells and leading to the defective recognition of the tumor [82], and TGF-β may be playing a role in this process [83]. NK cell function is also regulated by the crosstalk between immune cells in the TME. Multiple immune cell subpopulations, such as the myeloid derived suppressor cells (MDSC), T regulatory cells (Tregs), macrophages polarized to the immunoregulatory phenotype (M2), and immature Dendritic cells (DC) facilitate NK cell disfunction. MDSC and Treg may act on NK cells via membrane-bound TGF-β [84,85]. In addition to their direct effect on NK cells, myeloid cells [86] as well as on Tregs [87], TGF-β signaling has been demonstrated as a critical mediator in tumor invasion and metastasis through the action of tumor associated macrophages (TAM) that secrete growth factors, including TGF-β, which promote migration of endothelial cells and angiogenesis [88,89]. 

TGF-β in adaptive immune cells in cancer: The effect of TGF-β in the adaptive immune response against HCC is very broad and affects to all T lymphocyte subpopulations. TGF-β produced by cancer cells can induce an immature differentiation state of DC, converting them into tolerogenic DC [90] with a downregulated expression of MHC class-II molecules [91], impaired cross-presenting capacities and downregulated costimulatory molecule expression [92]. These TGF-β-induced immature DC facilitate tumor tolerance by inducing antigen-specific CD8+ Tregs, suppressing the function of other effector T cells [92,93]. TGF-β can directly inhibit the cytotoxic functions of CD8 T cells [94]. On CD4 T cells, TGF-β affects the differentiation of both Th1 and Th2 subsets by downregulation of their key transcription factors [95,96,97] and ultimately favouring a shift of Th1 towards Th2 cell differentiation [98,99]. TGF-β affects T cell proliferation and effector functions by impairing IL-2 production during T cell activation [100], inducing cell cycle arrest and favouring apoptosis of T cells [101,102]. 

TGF-β activates SMAD2/3 and, in cooperation with IL-21 and IL-23, promotes the generation of Th17 cells contributing to NAFLD-associated liver inflammation and HCC development [103,104,105]. In CD8+ cytotoxic T cells, TGF-β cooperates with the transcription factor ATF1 to suppress the expression of IFN-γ to inhibit its antitumor activity [98]. Intratumoral TGF-β suppresses NKT cells, a population responsible for recruiting effector immune cells to the tumor through the production of large amounts of IFN-γ. Notably, the development of Invariant NKT (iNKT) cells, which represent a subclass of NKT cells with regulatory functions, is orchestrated by TGF-β [106], and plays an important role switching from inflammation to resolution of liver injury [107], but also may affect the outcome and overall survival in HCC [108,109].

The production of TGF-β, mainly by liver sinusoidal endothelial cells (LSEC) and HSC contribute to hepatic regulatory T (Treg) cell induction. There is a correlation between TGF-β and Treg cells in HCC patients. Moreover, the Treg-associated expression of both TGF-β and IL-10 was shown to be associated with HCC progression [110]. Both CD4+ Foxp3+ and CD4+ Foxp3- suppressor cells induced by TGF-β are increased in HCC patients and correlate with poor overall survival [111,112,113]. Notably, TGF-β can also elicit the production of other factors such as amphiregulin [114], that has a promoting role on the immunosuppressive activity of Treg cells [115,116]. The presence of Treg cells may play an important homeostatic role in tissue repair after injury. However, its immunosuppressive role may affect dramatically the antitumor activity of other immune cells. 

On the other hand, TGF-β1 enhances antigen-induced PD-1 expression through SMAD3-dependent transcriptional activation in antigen-specific T cells, suggesting that the TGF-β pathway directly participates in immune-checkpoint regulation [117].

All these data point to TGF-β as one of the master immunosuppressive molecules in HCC and imply that targeting the TGF-β pathway might enhance antitumor immunity in HCC patients.

## 5. TGF-β Inhibitors

**Figure 3:**
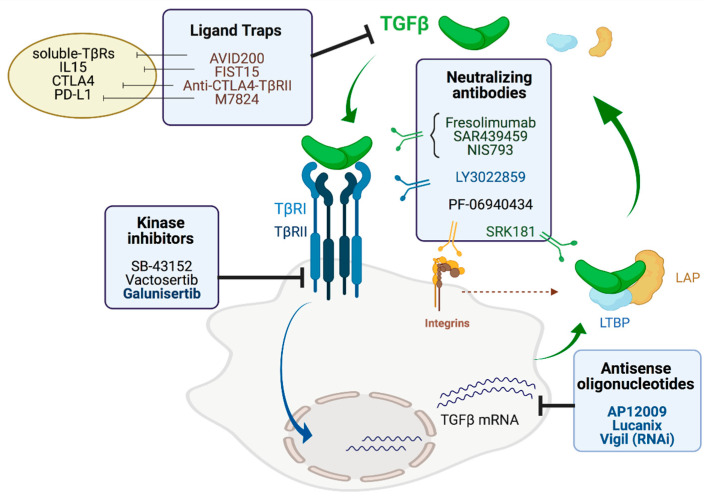
Schematic representation of the different strategies targeting TGF-β signaling for cancer therapy. According to their mechanism of action, directed against TGF-β expression, cytokine blockade or signaling inhibition, these strategies fall under different categories: (i) Antisense oligonucleotides; (ii) Neutralizing antibodies; (iii) Ligand traps; (iv) Small molecule inhibitors (kinase inhibitors). LAP: Latency Associated Peptide, LTBP: latent TGF-β binding proteins. Figure was created with BioRender.com.

The tumorigenic role of TGF-β in late-stage solid malignancies has spurred the development of a variety of anti-TGF-β drugs [11,118]. All the evidence discussed in previous sections indicates that targeting the TGF-β pathway may also constitute an effective strategy for HCC treatment in appropriately selected patients. Available TGF-β inhibitors are of different chemical nature and present diverse mechanisms of action including: (i) the suppression of the production of TGF-β; (ii) the inhibition of TGF-β activity; (iii) the blockage of the interaction of TGF-β with its receptors; and (iv) the inhibition of the kinase activity of the TGF-β receptor. These pharmacological effects may be achieved with antisense oligonucleotides, neutralizing antibodies, ligand traps and small molecule inhibitors (Figure 3). Many of these inhibitors have shown promising anti-tumoral activity in preclinical models, and an increasing number of them have been, or are currently being tested in clinical trials of almost all types of solid tumors, alone and in combination with other agents [11,118,119,120].

### 5.1. Antisense Oligonucleotides

Antisense oligonucleotides (AON) are designed to specifically bind target mRNAs and induce their degradation. AP12009 (Trabedersen) is an 18-mer AON targeting TGF-β2 mRNA that has been evaluated in clinical trials for several advanced solid tumors, including pancreatic and colorectal carcinoma, melanoma and glioma with encouraging results and a good safety profile [11,120]. Downregulation of TGF-β2 expression not only results in the inhibition of cancer cell growth, it may also enhance immunity against the tumor [121]. This notion led to the development of tumor cell vaccines like Lucanix, which expresses TGF-β2 AON, or Vigil that harbors a short hairpin RNAi targeting furin convertase, involved in TGF-β1 and TGF-β2 precursors processing [120]. These vaccines have been tested with promising results in patients with different solid tumors, such as lung, ovarian and metastatic Ewing’s sarcoma, but still not in HCC patients [120].

### 5.2. Neutralizing Antibodies

Several neutralizing monoclonal antibodies targeting binding of TGF-β to receptors have been developed and tested at different levels. After demonstrating the efficacy of the murine anti-TGF-β 1D11 antibody in preclinical in vivo models [122,123,124], the humanized version (Fresolimumab; GC-1008) was developed. It is a pan-neutralizing IgG4 antibody that binds to all three TGF-β isoforms and has been tested in the clinic in several neoplastic and non-neoplastic indications, as reviewed in [125], but not in HCC. In patients with melanoma or renal cell carcinoma [126] or with relapsed malignant pleural mesothelioma [127] therapy was well tolerated and a patient with complete response and several with stable disease were observed. Development of this antibody was discontinued and an improved version, SAR439459, has shown important immunomodulatory effects in preclinical in vitro and in vivo models [128]. SAR439459 has entered clinical trials as monotherapy in several solid tumors including melanoma, colorectal adenocarcinoma, urothelial cancer, HCC and non-small cell lung cancer (NCT03192345). In addition to their effects as monotherapy, this antibody improved immunogenicity and antitumor efficacy triggered by PD-1 blockade, suggesting its potential as partner in combinatorial immunotherapies [128]. Anti-PD-1-based therapies may enhance the Treg/CD4+ Th ratio and increase pSMAD3 expression in tumor cells. Since anti-TGF-β antibody administration attenuates these effects, the combined blockade of both molecules results in a synergistic effect, where additional immune-independent mechanisms are also targeted by this combination [129]. 

NIS793 is another anti-TGF-β antibody that is being tested in a phase I/Ib clinical trial in patients with different solid tumors (breast, lung, HCC, colorectal, pancreatic and renal) NCT02947165. In this case, as for other antibodies, it is being administered in combination with PD-1 blockade. 

Besides cytokine blockade, antibodies targeting the receptor have also been developed. LY3022859 is an IgG1 antibody that, upon binding to TβRII, prevents the formation of the ligand-receptor complex and thus inhibits the ensuing signaling activation. Preclinical data with this antibody showed antitumor effects mediated through several mechanisms [130]. However, results obtained in a clinical trial in patients with advanced solid tumors raised safety concerns related to uncontrolled cytokine release [131].

Other antibody-based strategies targeting additional TGF-β-related elements have been tested. The role played by some integrins in the activation process of surface TGF-β [132] suggested that anti-integrin antibodies would inhibit TGF-β activation, bypassing the effect of systemic TGF-β inhibition [133,134]. Indeed, expression of β8 integrin by cells that rely on TGF-β for their immunosuppressive effects, such as tumor cells [134] or Treg cells [135], controls some TGF-β-associated events, and blockade of this integrin results in antitumor activity in several preclinical murine models. In this regard, a clinical trial based on the administration of the αvβ8-blocking antibody PF-06940434 (NCT04152018) is testing this drug in several advanced and metastatic solid tumors (not including HCC). Interestingly, as for other inhibitory antibodies, some trial arms include a combination with anti-PD-1 antibodies. Finally, targeting of latent TGF-β1 and inhibition of its activation has been achieved with SRK-181 antibody. By considering the lower sequence similarity in the sequences of TGF-β1, 2, and 3 prodomains, this antibody was selected to specifically bind and block TGF-β1 activation [136]. Although monotherapy with this antibody did not provide any beneficial effect on tumor growth, combination with PD-1 blockade overcome resistance to this immunotherapy, associated to a profound remodeling of the tumor immune profile. These results have prompted a new clinical trial (NCT04291079) in patients with locally advanced or metastatic solid tumors, where SRK-181 is also considered in combination with PD-(L)1 blocking antibodies.

### 5.3. Ligand Traps

The basic rationale behind the use of ligand traps is to sequester the cytokine and avoid thus its binding to the receptors [137]. In the case of TGF-β, several compounds in this field have been developed [138,139,140,141] based on the use of soluble TβRII or III or receptors fused to immunoglobulins. AVID200 is a TGF-β1 and 3 inhibitor of this class [142,143] that has entered clinical trials (NCT03834662) in patients with advanced and metastatic malignancies.

In addition to molecules with TGF-β-binding domains, more complex molecules have been designed to enrich their number of functions, tackling thus different effector and/or immunosuppressive mechanisms. FIST15 is a molecule containing the IL15Rα-sushi domain bound to IL15 as well as the TβRII ectodomain [144]. FIST15 enhanced functional activity of CD8 T cells and NK cells, resulting in a higher antitumor effect in preclinical murine models. Resistance to checkpoint inhibitors has been associated with TGF-β expression [145,146]. Based on this, TGF-β ligand traps have been conjugated to PD-1/PD-L1 blocking molecules or to antiCTLA-4 [147,148]. These dual molecules have demonstrated superior preclinical antitumor efficacy compared with monotherapies blocking either TGF-β or the corresponding checkpoints, due to their capacity to activate innate and adaptive immunity. M7824 (Bintrafusp alfa) is being tested in many clinical trials in patients with a variety of solid tumors [149,150,151], including HCC [152]. In this last case, the phase I study showed a manageable safety profile and preliminary efficacy, warranting further assays in larger patient groups.

### 5.4. Small Molecule Inhibitors

The most explored strategy for TGF-β inhibition in cancer is based on small molecules that interfere with intracellular signaling from TGF-β receptors, suppressing canonical and non-canonical pathways. Preclinical studies with the TβRI kinase inhibitor SB-431542 demonstrated antitumoral activity in different tumors such as renal carcinoma and malignant glioma [120], and also in HCC [153,154]. Vactosertib (TEW-7197) is an orally available more potent and specific TβRI kinase inhibitor that has also shown efficacy in preclinical models of myeloma, breast and pancreatic carcinomas [119]. Vactosertib is currently undergoing clinical studies in patients with different solid tumors, with a favorable safety profile and its efficacy is being evaluated [119,120]. In vitro studies also showed inhibitory activity of Vactosertib in HCC cells growth [155]. Galunisertib (LY2157299) is another orally available drug and the most extensively studied of all small molecule inhibitors of TβRI kinase [156]. Galunisertib showed excellent antitumor activity in preclinical models of breast and colon cancer, among other solid tumors [120], and also demonstrated efficacy in HCC cell lines and experimental models [38,157,158]. Interestingly, Galunisertib treatment also increased the efficacy of Sorafenib on HCC cells growth inhibition and apoptosis [158,159]. Extensive preclinical and phase I studies led to the clinical development of Galunisertib, including the establishment of dosing strategies and its therapeutic window, and providing evidence of good tolerability [160]. The clinical efficacy of Galunisertib has been recently assessed in patients with advanced HCC who progressed on or were ineligible to receive Sorafenib [161]. This study showed that Galunisertib had a manageable safety profile, and that those patients in which circulating alpha fetoprotein (AFP) and TGF- β1 levels were reduced upon Galunisertib administration had longer survival (NCT01246986). A follow-up study from the same team confirmed the usefulness of plasma TGF-β1 levels as a biomarker to assess the clinical activity of this TβRI inhibitor [162]. Subsequent phase Ib and phase II studies demonstrated that the combination of Galunisertib and Sorafenib in patients with advanced HCC was well tolerated and prolonged overall survival [15,163,164]. Together, these studies underscore the potential of Galunisertib for advanced HCC treatment, and open the door for additional combinations of this drug with other antitumoral agents. 

## 6. Current Therapies in HCC and New Perspectives for TGF-β Inhibitors

The landscape of HCC has been substantially changed with the incorporation of Atezolizumab and Bevacizumab in 2020 as first-line treatment for advanced HCC [165]. The breakthrough impacted not only on the clinical decision-making but also into the clinical trials design. Until 2018 when the REFLECT trial (Lenvatinib vs. Sorafenib, no-inferiority design) was positive [166], the only first-line treatment was Sorafenib and all evidence-based data in second-line treatments were demonstrated in patients treated with Sorafenib as first-line treatment [167,168,169] (Several revisions describe these clinical trials characteristics and outcome [1,170,171]). However, despite currently having several options in clinical practice, the benefits of sequential HCC treatments vary across patients and its impact is not only related to the baseline tumor burden or related symptoms. Indeed, despite having predictors of outcome such as baseline BCLC stage, ECOG-PS, or AFP or evolutionary-events such us early-dermatologic adverse events in patient’s treatment with tyrosine-kinase inhibitors [172,173,174] that could be associated to better outcome, the rate of tumor progression and time to progression would be defined by factors that have not been identified yet. So, precision oncology aims to identify molecular factors that could help clinicians in their clinical practice at the time of defining the need of changing from one treatment-line to the other. 

In this regard, strong evidences suggest that an immunosuppressive TME may contribute to therapeutic failure. Therefore, it was no wonder that drugs that block the binding of PD-L1 to PD-1 were effective in the treatment of advanced HCC. Indeed, Nivolumab [175] and Pembrolizumab [176] have been granted an accelerated FDA approval in second-line treatment based on radiologic tumor response. However, despite the incredible clinical advance in HCC treatment represented by PD-1-PD-L1 interaction inhibitors, a recent phase III trial in second-line comparing the PD-1 inhibitor Pembrolizumab vs. placebo failed to improve overall survival (OS) and progression-free survival (PFS) [177]. Moreover, a phase III study of Nivolumab vs. Sorafenib in first-line treatment (Checkmate 459, NCT02576509) [178] showed that HCC patients treated with Nivolumab presented better median OS (16.4 months compared to 14.7 in the patients who received Sorafenib [HR 0.85 (95% CI; 0.72–1.02; 0.075)] although the results did not reach statistical significance. Therefore, despite the clinical and quality of life benefits of Nivolumab, this is another negative study. This deceiving failure of immunotherapy in HCC exposes the specific molecular characteristics of this cancer and the need to unravel the mechanisms of such resistance to treatment. Subsequently highlighting the urgent need for specific biomarkers which allow to identify patients that would benefit from therapy with immune checkpoint inhibitors. 

The rise of new drugs available in HCC prompts an attractive attempt to combine different approaches, such as the immune checkpoint inhibitors and targeted therapy with the aim of improving response and survival. Indeed, there are currently 4 phase III studies recruiting or pending to report results of combination treatments: LEAP-002 (Lenvatinib + Pembrolizumab vs. Lenvatinib) (NCT03713593), COSMIC-312 (Cabozantinib + Atezolizumab vs. Sorafenib) (NCT0329851), HIMALAYA (Duravalumab ± Tremelilumab vs. Sorafenib) (NCT03298451) and CheckMate 9DW (Nivolumab + Ipililumab vs. Sorafenib or Lenvatinib).

In this context, it is essential to keep in mind that the rationale for combinations relies not only on the additive therapeutic effect, but also on the potential immunomodulation property of target agents and their impact on the immunosuppressive TME. In this sense, targeting TGF-β1 is of particular interest due to its potent immunosuppressive effects thoroughly described above. However, none of the current phase I-II trials in advanced HCC (Table 2) or the ongoing phase III trials focus on TGF-β signaling, despite data from prior studies showing promising results of TGF-β modulation in HCC as previously mentioned. 

Indeed, Galunisertib has been tested as second-line monotherapy in 149 HCC patients in a phase II study (Table 3), predefining baseline AFP values as a biomarker (greater than 400 ng/mL) (NCT01246986) [162]. The median OS was 7.3 months for patients with AFP > 400 and 16.8 months for patients with AFP ≤ 400 ng/mL. In this study high baseline levels of plasma TGF-β1 and E-Cadherin have also associated with poor outcome and patients who decrease more than 20% from baseline in AFP and TGF-β1 levels had a significantly prolonged OS (21.5 months). Results of Galunisertib in combination with Sorafenib 400 mg BID were also reported in the same phase II clinical trial (NCT01246986) [164] (Table 3). The median TTP was 4.1 months and median OS was 18.8 months. Both were analyzed by baseline biomarker values: AFP < 400 ng/mL and ≥400 ng/mL and TGF-β1 less than or greater than/equal to baseline median. OS was significantly longer in TGF-β1 responders (decrease more than 20% from baseline) (22.8 vs. 12 months, *p* < 0.038). Moreover, Bintrafusp alfa, designed to simultaneously target TGF-β and PD-L1, has shown antitumor activity both as monotherapy and in combination with chemotherapy in preclinical studies. This drug previously designated M7824 was also evaluated in Asian patients with advanced solid tumors, including an HCC in a phase I, open-label, dose escalation study at different doses 3, 10, and 20 mg/kg, every 2 weeks (NCT02699515) which the primary objective was safety and tolerability and the secondary objective was best overall response. Authors concluded that the Bintrafusp alfa had a manageable safety profile and showed preliminary efficacy in data [152]. However, there were only 10 patients with HCC, 1 was screening failure and there is not data available from no-Asian patients. 

The promising survival observed for the combination of Galunisertib with Sorafenib supports further exploration of its potential in other combinatorial strategies, including combination with immune checkpoint inhibitors. In this regard, preclinical studies have shown the synergistic activity of Galunisertib and checkpoint inhibitors in different tumor types [179], and a clinical trial is testing the combination of Galunisertib and Nivolumab in patients with solid tumors, including HCC (NCT02423343). In addition, Reiss et al. found that Galunisertib combined with Stereotactic Body Radiotherapy (SBRT) is well tolerated and associated with antitumor activity in patients with HCC [180]. Besides Galunisertib and Bintrafusp alfa, two TGF-β neutralizing antibodies are currently under evaluation in two independent ongoing clinical trials (NCT03192345 and NCT02947165) in combination with immune checkpoint inhibitors in HCC (Table 3).

Further research is needed to translate the current knowledge of HCC biology to prognostic and predictive biomarkers in order to guide clinical decision to improve patient outcomes. In this regard, analyzing the molecular landscape of tumor samples obtained from patients starting first line and also upon progression prior to second line or to third line is crucial to identify resistance signatures of signaling pathways and ultimately, design novel therapeutic strategies using a personalized decision-making pipeline.

## 7. Concluding Remarks

TGF-β pathway is difficult to target, considering that its inhibition in the wrong patients could do more harm than good. However, there is no doubt about its potential as a therapeutic option in HCC, due to the strong pro-tumorigenic effects that TGF-β might mediate at later stages in the tumor cell and at all the stages in the liver tumor stroma. Furthermore, TGF-β could favor immune evasion and is an interesting target to inhibit in case of immunotherapy approaches. Nevertheless, to efficiently target the TGF-β pathway, it is mandatory to deepen into the molecular mechanisms through which TGF-β promotes tumor progression as well as to identify relevant biomarkers of the TGF-β oncogenic arms.

## Figures and Tables

**Table 1:** TGF-β signature and biomarkers in human HCC cell lines.

Cell Line	Tumor Type	Phenotype	TGF-β Signature	Biomarkers (Expression Levels)					
				CDH1	VIM	CD44	CXCR4	SMAD7	CLTC
HepG2	Human caucasian HCC	Epithelial	Early	High	Absent	Absent	Low	Low	No data
PLC/PRF/5	Human liver hepatoma	Epithelial	Early	High	Very Low	Low	Low	Low	Low
Huh-7	Human asian HCC	Mixed	Early	Medium	Low	Low	Low	High	Low
Hep3B	Human black HCC	Mixed	Early	Medium	Low	Low	High	High	Low
SNU-449	Human asian HCC	Mesenchymal	Late	Absent	High	High	High	No data	High
HLE	Human HCC	Mesenchymal	Late	Absent	High	High	High	High	high
HLF	Human HCC	Mesenchymal	Late	Absent	High	High	High	High	High

CDH1: E-Cadherin, CLTC: Clathrin, Vim: Vimentin.

**Table 2:** Current phase I-II trials in intermediated/advanced HCC.

No. Clinical Trial	Population	Arms	N	Aim	Status
NCT01988493	Advanced	Tepotinib vs. Sorafenib	117	Safety and TTP	Active, no Recruiting
NCT03970616	Advanced	Tivozanib + Durvalumab (1st Line)	42	Safety	Recruiting
NCT04503902	Advanced	Donafenib Tosilate + Toripalimab	46	Safety and ORR	Not yet Recruiting
NCT04612504	Advanced	SynOV1.1 monotherapy vs. SynOV1.1 + Atezolizumab	45	Safety	Not yet Recruiting
NCT03825705	Advanced	TQB2450 injection + Anlotinib	60	ORR	Recruiting
NCT03893695	Intermediate (not locoregional)/Advanced	GT90001 + Nivolumab (dose escalation and expansion)	20	Safety	Active, no Recruiting
NCT03864211	Intermediate (not locoregional)/Advanced	Toriplimab montherapy vs. toriplimab + Ablation	130	PFS	Recruiting
NCT04502082	Intermediate (not locoregional)/Advanced	ET140203 autologous T cell product (3rd Line)	50	Safety	Recruiting
NCT03998033	Intermediate (not locoregional)/Advanced	ET140202 autologous T cell product	50	Safety	Active, no Recruiting
NCT04035876	Intermediate	Camrelizumab + Apatinib (Downstaging for TOH)	120	ORR and RFS	Recruiting
NCT04069949	Advanced	Toripalimab + Sorafenib (1st Line)	39	Safety and 6-month PFS	Not yet Recruiting
NCT03897543	Intermediate (not locoregional)/Advanced	ABX196 + Nivolumab (2nd Line)	48	Safety	Recruiting
NCT04212221	Intermediate (not locoregional)/Advanced	MGD013 monotheray vs. MGD013 + Brivanib (2nd Line)	300	Safety	Recruiting
NCT04601610	Advanced	KN046 + Ningetinib (1st or 2nd Line)	70	Safety and ORR	Not yet Recruiting
NCT04380545	Intermediate (not locoregional)/Advanced	Fluorouracil + Nivolumab + Recombinant Interferon Alpha 2b-like protein	15	Safety	Not yet Recruiting
NCT03941873	Intermediate (not locoregional)/Advanced	Sitravatinib monotherapy vs. Sitravatinib + Tislelizumab (1st o 2nd Line)	104	Safety and ORR	Recruiting
NCT03812874	Intermediate	PTX-9908 + TACE vs. PBO + TACE	50	Safety	Recruiting
NCT04251117	Intermediate (not locoregional)/Advanced	GNOS-PV02 + INO-9012 + Pembrolizumab (2nd Line)	24	Safety and Immunogenicity of a personalized neoantigen DNA vaccine.	Recruiting
NCT03941626	Unresectable HCC/no effective treatment	CAR-T/TCR-T cells immunotherapy	50	Safety	Recruiting
NCT03829501	Advanced	KY1044 monotherapy vs. KY1044 + Atezolizumab	412	Safety	Recruiting

TTP: time to progression, ORR: overall response rate, PFS: progression- free survival, RFS: recurrence free survival, TACE: transarterial chemoembolization, PBO: placebo.

**Table 3:** Clinical trials evaluating TGF-β inhibitors in advanced HCC.

No. Clinical Trial	Population	Phase	Arms	N	Aim	Status
NCT01246986 (Parts A and B)	Advanced	II	Galunisertib	149	Association of circulating AFP and TGF-β1 levels with OS	Completed
NCT01246986 (Part C)	Advanced		Galunisertib + Sorafenib	47	Safety, TTP, OS, PFF and ORR	Completed
NCT02240433	Unresectable	I	Galunisertib + Sorafenib	14	Safety, tolerability, PK, TTP and PFS	Completed
NCT02906397	Advanced	I	Galunisertib + SBRT	15	Safety, PFS, OS	Active, no Recruiting
NCT02423343	Advanced	I/II	Galunisertib + Nivolumab	75 (10 HCC)	Safety, tolerability, PFS, ORR	Completed
NCT02699515	Advanced	I	Bintrafusp alfa MSB0011359C (M7824)	114	Safety, tolerability and ORR	Active, no Recruiting
NCT03192345	Advanced	I (Basket trial)	SAR439459 monotherapy vs. SAR439459 + Cemiplimab	~350	Safety, tolerability, TTP and PFS	Recruiting
NCT02947165	Advanced	I (Basket trial)	NI5793 monotherapy vs. NI5793 + PDR001	120	ORR and PFS	Active, no Recruiting

TTP: time to progression, ORR: overall response rate, OS: Overall Survival, PFS: progression-free survival, PK: Pharmacokinetics, SBRT: Stereotactic Body Radiotherapy.
